# Rapid Cycling Genomic Selection in a Multiparental Tropical Maize Population

**DOI:** 10.1534/g3.117.043141

**Published:** 2017-05-22

**Authors:** Xuecai Zhang, Paulino Pérez-Rodríguez, Juan Burgueño, Michael Olsen, Edward Buckler, Gary Atlin, Boddupalli M. Prasanna, Mateo Vargas, Félix San Vicente, José Crossa

**Affiliations:** * Global Maize Program, International Maize and Wheat Improvement Center (CIMMYT), 06600 México D.F., México; † Colegio de Postgraduados, CP 56230, Montecillos, 56230 México D.F., México; ‡ Biometrics and Statistics Unit, International Maize and Wheat Improvement Center (CIMMYT), 06600 México D.F., México; § International Maize and Wheat Improvement Center (CIMMYT), Nairobi 1041-00621, Kenya; ** United States Department of Agriculture, Agricultural Research Service, Cornell University, Ithaca, New York 14853; †† Bill and Melinda Gates Foundation, Seattle, Washington 98109; ‡‡ Universidad Autónoma Chapingo, 56230 Texcoco, México

**Keywords:** tropical maize, multiparental population, rapid cycling recombination genomic selection, realized genetic gains, genetic diversity, Multiparental Populations MPP

## Abstract

Genomic selection (GS) increases genetic gain by reducing the length of the selection cycle, as has been exemplified in maize using rapid cycling recombination of biparental populations. However, no results of GS applied to maize multi-parental populations have been reported so far. This study is the first to show realized genetic gains of rapid cycling genomic selection (RCGS) for four recombination cycles in a multi-parental tropical maize population. Eighteen elite tropical maize lines were intercrossed twice, and self-pollinated once, to form the cycle 0 (C_0_) training population. A total of 1000 ear-to-row C_0_ families was genotyped with 955,690 genotyping-by-sequencing SNP markers; their testcrosses were phenotyped at four optimal locations in Mexico to form the training population. Individuals from families with the best plant types, maturity, and grain yield were selected and intermated to form RCGS cycle 1 (C_1_). Predictions of the genotyped individuals forming cycle C_1_ were made, and the best predicted grain yielders were selected as parents of C_2_; this was repeated for more cycles (C_2_, C_3_, and C_4_), thereby achieving two cycles per year. Multi-environment trials of individuals from populations C_0,_ C_1_, C_2_, C_3_, and C_4_, together with four benchmark checks were evaluated at two locations in Mexico. Results indicated that realized grain yield from C_1_ to C_4_ reached 0.225 ton ha^−1^ per cycle, which is equivalent to 0.100 ton ha^−1^ yr^−1^ over a 4.5-yr breeding period from the initial cross to the last cycle. Compared with the original 18 parents used to form cycle 0 (C_0_), genetic diversity narrowed only slightly during the last GS cycles (C_3_ and C_4_). Results indicate that, in tropical maize multi-parental breeding populations, RCGS can be an effective breeding strategy for simultaneously conserving genetic diversity and achieving high genetic gains in a short period of time.

In the last 20 yr, marker-assisted selection has been widely used in plant breeding where a few markers significantly associated with the phenotypic trait are employed to predict the genetic value of the candidates for selection (Bernardo 2008, 2016). On the other hand, genomic-assisted breeding (genomic selection, GS) incorporates all available marker information simultaneously into a model to predict the genetic value of the candidates for selection (Meuwissen *et al.* 2001). In plants, a computer simulation study (Bernardo and Yu 2007) showed that better prediction accuracy of breeding and genetic values was achieved by incorporating all markers, as compared to using a subset of markers significantly associated with QTL. This result was verified by Massman *et al.* (2013), who used a biparental temperate maize population derived from a cross between two distinct heterotic groups (B73 and Mo17); the testcrosses were evaluated under well-watered conditions, and the population advanced using rapid cycling GS (RCGS where all markers are used for prediction) and marker-assisted recurrent selection (MARS where only significant markers are used for prediction). Massman *et al.* (2013) reported that RCGS had a superior response for stover yield, as well as stover and grain yield indices that were 14–50% higher than those of MARS.

In tropical maize, Beyene *et al.* (2015) evaluated realized genetic gains in grain yield from RCGS in eight biparental maize populations in drought stress environments. The authors found that the average gain from RCGS per cycle across eight populations was 0.086 ton ha^−1^ and that hybrids derived from cycle 3 produced 7.3% (0.176 ton ha^−1^) higher grain yield than those developed through the conventional pedigree breeding method. RCGS in biparental populations offered the advantage of significant time efficiency over conventional breeding methods, as up to three cycles of RCGS can be conducted within a year. Interestingly, Beyene *et al.* (2015) pointed out that the average genetic gain per year in tropical maize grain yield using RCGS was three times higher than that achieved by using conventional pedigree-based phenotypic selection in drought stress environments. Furthermore, Vivek *et al.* (2016) recently reported a study on realized genetic gains in grain yield using two biparental populations generated by crossing two elite Asian maize inbred lines with an African drought tolerant line. Cycle 1 (C_1_) was formed by recombining the top 10% of the F_2:3_ families. Cycle 2 (C_2_) was derived using two different methods: (1) phenotypic selection (C_2_-PS); and (2) GS (C_2_-GS). Results showed that C_2_-GS top-crosses produced 4–43% higher grain yield than C_2_-PS top-crosses.

For RCGS within biparental populations, prediction accuracy is achieved thanks to high linkage disequilibrium (LD), no pedigree, and no family substructure (Crossa *et al.* 2014; Zhang *et al.* 2015). However, predictions across biparental populations will be poor if unrelated biparental populations with different allelic diversity are used as the training population. Furthermore, Jannink *et al.* (2010) outlined some of the disadvantages of using GS applications in biparental populations: (1) separate model training is required for each biparental cross; GS should be applied to the entire population; and (2) the first generation of progeny from a cross needs to be phenotyped and candidates cannot be selected on the basis of prior information, a practice that slows down the breeding cycle. Biparental populations have also been widely used for detecting and mapping QTL. However, QTL mapping power and resolution might be comparatively reduced in biparental mapping populations as the number of segregating causal loci is very low, because large blocks of parental chromosomes are preserved (Cavanagh *et al.* 2008; Thepot *et al.* 2015; Lehermeier *et al.* 2014). The problem of limited allelic diversity in one genetic background which occurs in biparental populations can be overcome by the use of multi-parental populations (MPP) with greater allelic diversity and from different genetic backgrounds (Verhoeven *et al.* 2006), along with increased polymorphism and recombination as compared to biparental populations (Ahfock *et al.* 2014).

Different MPPs have been constructed with the aim of increasing precision in fine mapping. For example, researchers have combined different biparental populations using factorial crosses, partial or complete diallels (Rebai and Goffinet 1993), or circular crosses (Cavanagh *et al.* 2008; Huang *et al.* 2012). The designs and analyses of these types of populations have been extended to genomic-enabled studies using different schemes and designs for efficient training, and for testing MPPs in different species.

Multi-parental populations are useful for mapping extensive numbers of loci. Thepot *et al.* (2015) used a multi-parental mapping population created after 12 generations of recombination among 60 founder wheat lines. The 12 generations of recombination broke up the LD and the existing population structure of the original populations. This approach to fine mapping helped to identify 26 genomic regions, six of which carried flowering QTL, and allowed detecting loci under selection and association mapping. In a recent article, Hoffstetter *et al.* (2016) used 47 bi-parental crosses, including 23 parental wheat lines as a training set, and 17 half-sib lines as a validation set, with some of the lines from the training set included in the testing set. Prediction accuracy using subsets of the training population to predict the validation sets ranged from 0 to 0.85. In maize, Lehermeier *et al.* (2014) evaluated prediction accuracy in 21 biparental doubled haploid populations, including 10 dent kernel type related populations, and 11 flint kernel type related populations. The authors found that prediction combining several half-sibs gave similar or higher prediction accuracy than predictions within biparental populations.

However, several theoretical complexities and challenges arise when attempting to perform GS in MPP. Plant breeders usually work with sets of full-sib families generated from crosses of inbred parents that vary in size so that extensive LD exists within each family; however, different LD patterns must exist across families. Therefore, because, in a typical MPP, the breeder is faced with LD across a large set of families, the level of LD as well as the marker density are important factors to be considered when applying GS to MPP. Zhong *et al.* (2009) using marker data on 42 two-row spring barley inbred lines simulated MPP with high and low LD populations generated from multiple inbred crosses. The authors suggested a trade-off between the model-method’s ability to capture LD between marker and QTL *vs.* its ability to exploit marker-based relatedness of individuals. Genomic relationship information (Best Linear Unbiased Predictor, GBLUP) is more valuable than LD information given by models that use marker effects. However, when markers were in strong LD with QTL with large effects, models based on marker effects were better predictors than models based on the genomic relationship between individuals.

The reliability of GS is greatly influenced by the number of phenotypes; therefore, combining data sets from MPP should increase the reliability of GS by making it more efficient and attractive for use in breeding. However, when combining populations, allele frequencies, LD and segregating haplotypes are different in different populations. Thus, when the marker effects are different between the different combined populations, this can reduce the reliability of GS. de Roos *et al.* (2009) studied this problem by combining two simulated cattle populations that diverged for certain number of generations; the authors found that increased genomic accuracy is achieved when all populations are combined in one training population but increasing marker density is required when the diversity of the combined population increases.

Despite the above theoretical and simulation results, which show that genomic-enabled prediction accuracy of MPPs is higher than the accuracy achieved within a single population, studies implementing RCGS in MPPs have not been reported. The research presented in this paper was initially conceived in 2009 and started in 2010. It included an initial MPP made up of 18 elite tropical maize lines intercrossed twice and self-pollinated once to form the cycle 0 (C_0_) training population. A total of 1000 ear-to-row C_0_ families were genotyped with dense GBS markers, and their testcrosses were phenotyped at four locations in Mexico to develop genomic prediction models. One cycle of phenotypic selection (C_0_–C_1_) and three cycles of RCGS (C_1_–C_4_) were carried out. The main objectives of this study were: (1) to report the realized genetic gains of four cycles (C_1_, C_2_, C_3_, and C_4_), plus the original training population (C_0_) in multi-environmental field trials of RCGS-assisted breeding evaluated together with four benchmark checks in two Mexican environments (locations), and (2) to investigate the genetic diversity of the families within each RCGS selection cycle to assess the level of genetic diversity after three cycles of rapid cycling GS.

## Materials and Methods

### Developing the training population from 18 tropical maize inbred lines

The RCGS experiment was designed in 2009 as part of the MasAgro project funded by Mexico’s Secretariat of Agriculture, Livestock, Rural Development, Fisheries and Food (SAGARPA, its acronym in Spanish) through the Sustainable Modernization of Traditional Agriculture program (MasAgro; http://masagro.mx).

**Figure 1:**
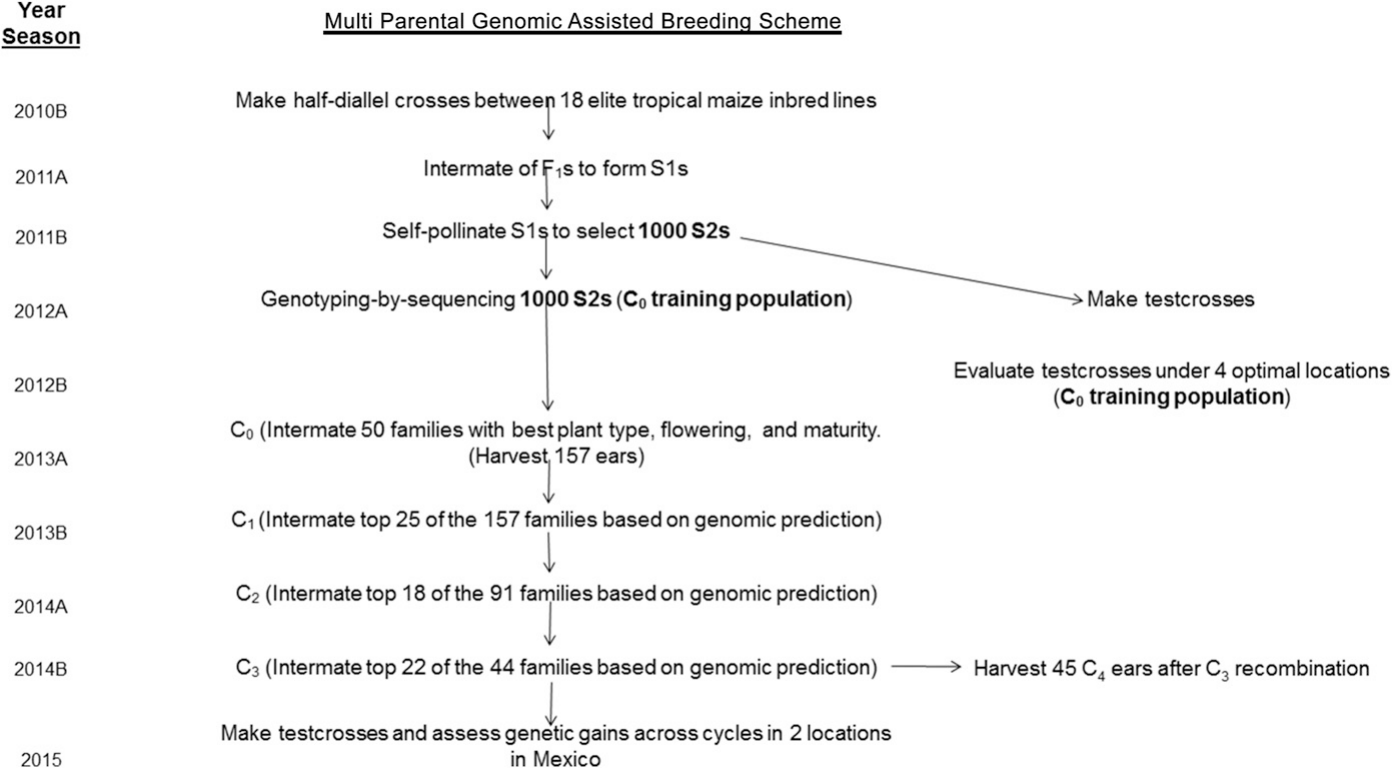
Breeding scheme used in the MPPs reported in this study.

The steps in the breeding scheme used for RCGS are shown in Figure 1. In total, 18 CIMMYT tropical maize inbred lines (CML247, CML264, CML448, CML494, CML498, CML531, CLRCW72, CLRCW75, CLRCW76, CLRCW93, CLRCW100, CLRCW260, CLWN201, CLWN228, CLWN229, CLWN247, CLG2312, and CLSPLW04), widely used in lowland tropical breeding environments, were crossed as parents to form the training population through twice intermated pollination and one self-pollination; the parents were selected based on their general combining ability for grain yield and *per se*, visual evaluation information for major stress tolerance and disease resistance in lowland tropical breeding environments. All 18 original parents tended to group in heterotic pattern group “B” (flint type kernel).

In the 2010B season, half-diallel crosses were made between the 18 original parents to generate all possible F_1_ progenies (Figure 1). In the 2011A season, all F_1_ were planted ear-to-row and intermated to form the S_1_ population; then, all the F_1_ were separated into two groups of equal size. Bulk pollen from the first group was used to pollinate all plants of the other group and vice versa; three ears were harvested from each F_1_ family, and equal amounts of seed from each selected F_1_ ear were bulked to form the subsequent generation for planting. In the 2011B season, 4800 S_1_ individuals were planted and self-pollinated and advanced to S_2_. The best 1000 S_2_ ears were selected, and planted ear-to-row in the 2012A season (Table 1). A single-cross tester (CML495/CML549) from the complementary heterotic group (dent type kernel) was used to generate testcrosses. All pollination activities were conducted at CIMMYT’s experiment station in Agua Fria, Puebla.

**Table 1:** Number of families and individual plants sown, selected, and advanced in each breeding cycle and among-family, within-family, and total selection intensity

	Cycle				
	C_0_	C_1_	C_2_	C_3_	C_4_
No. of families sown	1000	157	91	44	–
No. of families selected	50	25	18	22	–
No. of plants sown per family	25	25	25	25	–
No. of ears selected	157	91	44	22	45
Among-family selection intensity	5%	16%	20%	50%	–
Within-family selection intensity	12.60%	2.30%	1.90%	2.00%	–
Total selection intensity	0.60%	0.40%	0.40%	1.00%	–

The training population (C_0_) for developing genomic prediction models was formed with the best 1000 selected S2s. Testcrosses of all 1000 selected S_2_ were planted using a partial replicated design with 25% of replicated genotypes at four optimal Mexican locations. Phenotypic data were collected at all locations for >10 agronomic traits, including grain yield at 12.5% moisture content (GY), anthesis date (AD), silking date (SD), plant height (PH), ear height (EH), and moisture content (MOI). For each S_2_ family, DNA was extracted by bulking equal amounts of leaf tissue from 15 individual plants. Genotyping-by-sequencing was performed at Cornell University Biotechnology Resource Center as described by Wu *et al.* (2016), where 955,690 SNPs were generated for each DNA sample. In the training population, the genomic prediction model was developed by using only 331,740 filtered SNPs with minor allele frequency (>0.05), and where the missing data rate was <10%.

### Cycle 0 (C_0_) phenotypic selection and formation of cycle 1 (C_1_)

In C_0_, phenotypic selection was conducted by ranking the grain yield of the 1000 S_2_ testcrosses. The best 50 respective S_2_ families were selected and planted ear-to-row, 25 plants per family (Table 1). Cycle 1 (C_1_) was formed by intermating the 50 selected S_2_ families. The 50 families were divided into two equal groups, and bulk pollen from the first group was used to pollinate all plants in the other group and vice versa. Based on visual evaluation of flowering time, plant type, plant/ear height, well-filled ears, and reaction to naturally occurring major diseases, along with among-family and within-family selection, 157 ears (1–6 ears from each selected family) were harvested and shelled individually to form C_1_.

### Rapid cycling recombination of GS cycle 1 (C_1_), cycle 2 (C_2_), and cycle 3 (C_3_)

In C_1_, 157 selected ears (Table 1) were planted ear-to-row, 25 plants per family. DNA was extracted from the bulk tissue and shipped to Cornell University Biotechnology Resource Center for genotyping-by-sequencing. The Genomic Best Linear Unbiased Predictor (GBLUP) model (VanRaden 2007, 2008) implemented in the BGLR package was used for genomic prediction. Genomic estimated breeding values were calculated for all 157 C_1_ families; among-family selection was implemented based on genomic estimated breeding value information. The top 25 families were selected and intermated to form the C_2_ population. The 25 families selected were divided into two equal groups. Bulk pollen from one group was used to pollinate all plants in the other group and vice versa. Within-family selection was implemented based on visual evaluation of flowering time, plant type, plant/ear height, well-filled ears, and reaction to naturally occurring major diseases. A total of 91 ears were harvested, and shelled individually to form C_2_. In C_2_ and C_3_, the recombination protocol was repeated. The number of families and individual plants planted per cycle; number of families selected for next cycle recombination; number of ears harvested per cycle; and selection intensity information are listed in Table 1. After C_3_ recombination, 45 ears were harvested and shelled individually to form C_4_; within-family selection was implemented based on visual evaluation of agronomic traits. Families from Cycle 4 (C_4_) were not genotyped, as this was the last rapid cycling recombination.

### Phenotypic evaluation of the selection cycles for assessing genetic gains—benchmark checks and experimental designs in multi-environment trials

A total of 233 testcrosses was tested in the field to estimate the realized genetic gains; these entries belong to different groups. One group of entries (48) represents selection cycle C_0_, which is a subset of the best 50 families selected from the training population. Another group of entries represents RCGS cycles C_1_ (47 entries), C_2_ (48 entries), C_3_ (43 entries), and C_4_ (43 entries), and the last group of entries comprises the four benchmark checks (two local checks, one commercial check, and one experimental baseline check formed by testcrossing all 18 original parents with a single-cross tester (CML495/CML549). All the entries were crossed with a single-cross tester (CML495/CML549). Their testcrosses were planted at two locations in Mexico (Agua Fria and Tlaltizapan) in a modified split-plot design where the selection cycles were the main plots and the entries within each selection cycle were the subplots. The experimental design within each selection cycle (main plot) was an alpha-lattice design with two replications per location. The four benchmark checks were repeated in each subplot and planted together with the entries belonging to the different GS cycle; for example, the four checks were planted in the two replicates of the subplot where entries from cycle C_0_ were planted.

### Statistical analyses

Phenotypic data were collected at the two locations for the main agronomic traits including GY, AD, SD, PH, EH, and MOI. A linear mixed model was fitted to the data considering the incomplete block within replicate as random effects, and locations, cycles (main plot), entry within cycle (subplot), cycle × location interaction, and entry within cycle × location interactions as fixed effects. Random errors are assumed to be identically and independently normally distributed with mean zero and homogeneous variance. ANOVA for GY were performed including the evaluated entries from the selection cycles and the checks. Genetic gain response was assessed by regressing mean GY values on the selection cycle means (C_0_, C_1_, C_2_, C_3_, and C_4_) within each location and combined across both locations. ANOVA for AD, SD, PH, EH, and MOI were performed for each location and combined across both locations.

### SNP genotyping

For each cycle, bulk DNA of each planted family was sent to the Biotechnology Resource Center of Cornell University for genotyping-by-sequencing. The number of DNA samples used for genotyping were 1000, 157, 91 and 44 in C_0_, C_1_, C_2_ and C_3_, respectively. Families from C_4_ were not genotyped. Genotyping-by-sequencing, SNP calling, imputation and filtering were performed as described by Wu *et al.* (2016). Briefly, genomic DNA was digested with the restriction enzyme *Ape*KI. GBS libraries were constructed in 96-plex and sequenced on Illumina HiSeq2000 (Elshire *et al.* 2011). SNP calling was performed using the TASSEL GBS Pipeline, and a GBS 2.7 TOPM (tags on physical map) file was used to anchor reads to the Maize B73 RefGen_v2 reference genome (Glaubitz *et al.* 2014). Imputation was carried out with the FILLIN method in TASSEL 5.0 (Swarts *et al.* 2014), which anonymized GBS 2.7 haplotypes made from 8000-site windows. In total, 955,690 SNPs were generated for each sample, filtering was performed with minor allele frequency (>0.05) and the missing data rate was <10%.

### Assessing the genetic diversity of the selection cycles

Based on genomic data, we computed two genetic diversity indices between the families of the different selection cycles as well as the 18 parents. We calculated the Shannon Diversity Index of the sample for each selection cycle as $\frac{1}{A}\sum_{a=1}^{A}{\hat{p}}_{a}\text{ln}\left({\hat{p}}_{a}\right),$ where ${\hat{p}}_{a}$ is the frequency of the major allele in the *a*th marker over the entire sample, and A is the total number of markers. The expected proportion of heterozygous loci per individual was computed as the mean of heterozygosity for each marker as $0\leq \frac{1}{L}\sum_{l=1}^{L}\left(1-\sum_{a=1}^{n_{i}}{\hat{p}}_{la}^{2}\right)\leq 1$, where ${\hat{p}}_{la}$ is the frequency of the major allele in the *a*^th^ marker of the *l*^th^ individual, and *L* is the number of individuals.

Multidimensional scaling (MDS) was performed with the TASSEL software (http://www.maizegenetics.net/tassel) to assess the genetic similarity of all the materials in each selection cycle.

### Data availability

The phenotypic and genotypic data for the training population (cycle C_0_) evaluated in four sites, the phenotypic and genotypic data for the evaluation of the entries from the different selection cycles (C_0_, C_1_, C_2_, C_3_, and C_4_), as well as a brief GUIDE can be found in the link http://hdl.handle.net/11529/10927. A marker information file and characteristics of the genetic materials are also included in the link.

## Results

### Heritability and prediction accuracy of GY in the training population

The combined GY heritability across both locations was 0.34, while GY heritability at individual locations was 0.48, and 0.19 in Agua Fria and Tlaltizapan, respectively. Low-to-intermediate GY heritability was observed in the individual location analysis and combined analysis, mainly because GY is a complex trait. To mimic future prediction problems we will face, we implemented a fivefold random cross-validation with 100 replicates using entries in C_0_ (training population) for GY; the mean correlation between the predicted and observed values was 0.55.

### Realized genetic gains from rapid cycling recombination of GS for grain yield

A total of five groups of entries from C_0_, C_1_, C_2_, C_3_, and C_4_ plus four checks were used for field evaluation at two Mexican locations (Agua Fria and Tlaltizapan). Mean grain yield for each cycle and average gains per cycle are shown in Table 2. The Tlaltizapan location had the highest mean yield, with C_4_ reaching 10.96 ton ha^−1^. At both individual locations, the average performance across all C_4_ entries surpassed the grain yield performance of the other cycles; average grain yield performance across all C_4_ entries was 7.13 ton ha^−1^, and 10.96 ton ha^−1^ for Agua Fria and Tlaltizapan, respectively. Also, the combined analyses of the two locations showed an increase in mean grain yield in C_4_ of 9.05 ton ha^−1^ over that achieved in C_3_ (8.92 ton ha^−1^) and over the other GS cycles.

**Table 2:** Mean of GY (ton ha^−1^) for each genomic cycle C_0_, C_1_, C_2_, C_3_, and C_4_, broad-sense heritability (H^2^), and mean of the four testers at each location (Agua Fria and Tlaltizapan), and combined across the two locations

Cycle	Agua Fria			Tlaltizapan			Combined		
	Entry	H^2^	Checks	Entry	H^2^	Checks	GY	H^2^	Checks
C_0_	6.65	0.27	5.47	10.40	0.65	8.08	8.52	0.42	6.77
C_1_	6.49	0.06	5.73	10.29	0.59	9.32	8.40	0.63	7.52
C_2_	7.02	0.26	6.02	10.20	0.46	9.30	8.62	0.47	7.52
C_3_	6.88	0.38	5.64	10.95	0.59	9.31	8.92	0.67	7.52
C_4_	**7.13**	0.21	5.70	**10.96**	0.25	9.30	**9.05**	0.43	7.61
LSD_0.05_ (C_0_–C_4_)	0.402	—	—	0.412		—	0.252		—
LSD_0.05_ (C_1_–C_4_)	0.408	—	—	0.404		–	0.191		—
Average gain per cycle (C_0_–C_4_)	0.131	—	—	0.177		—	0.158		—
Average gain per cycle (C_1_–C_4_)	0.171	—	—	0.276		—	0.225		—

The average genetic gain in GY across cycles was estimated for each location and across both locations including all selection cycles (C_0_–C_4_), and including only the genomic selection cycles (C_1_–C_4_). Least significant differences (LSD) test at the 0.05 probability level including all selection cycles (C_0_–C_4_) and only the genomic selection cycles (C_1_–C_4_). The highest value is indicated in boldface.

For each location and combined, the entries representing the first selection cycle (C_1_) had lower GY than entries representing the base selection cycle (C_0_); this was due to the fact that the parents of the C_1_ population (Table 1) were not selected based on the grain yield performance of the testcrosses *per se*; instead, among- and within-family selection was conducted based on visual evaluation for flowering time, plant type, plant/ear height, well-filled ears, and reaction to naturally occurring major diseases. The other important reason is that the best 50 selected families were used to represent selection cycle C_0_ in the genetic gain evaluation study (rather than the random selected families). In C_3_, GY substantially increased in TL and combined across two locations with respect to previous selection cycles. For analyses in Agua Fria location, GY declined slightly to 6.88 ton ha^−1^ for C_3_ (Table 2). However, in the subsequent genomic selection cycles, the increases in realized genetic gains are clear for each location and combined across locations.

All selection cycles had higher average grain yield than the corresponding mean of the checks for individual locations and combined. Concerning broad-sense heritability, there was a decline in cycles C_1_ and C_4_ as compared to the base cycle (C_0_) in Agua Fria and Tlaltizapan; however, the opposite occurred when combining cycles C_1_ and C_3_ in both locations as compared to the heritability in the base cycle (C_0_). In the combined analyses across both locations, broad-sense heritability (H^2^) showed no decrease. In general, results showed that at each location and combined, while there was a decrease in GY from C_0_ to C_1_ there were also important increases in realized genetic gains for trait GY at the two locations from C_2_ to C_3_ and from C_3_ to C_4_. The mean grain yield of the benchmark checks varied slightly for each cycle at Agua Fria but stayed fairly constant at Tlaltizapan and combined locations for all selection cycles. Mean performance of the checks in selection cycle C_0_ was consistently lower than the mean performance in the other selection cycles.

The average gains per cycle for each location and combined across the two locations ranged from 0.131 ton ha^−1^ to 0.177 ton ha^−1^ when considering all cycles (C_0_–C_4_), and from 0.171 ton ha^−1^ to 0.276 ton ha^−1^ when considering only rapid cycling GS (C_1_–C_4_). For the combined location analyses, the realized genetic gains were 0.158 ton ha^−1^ and 0.225 ton ha^−1^ for cycles (C_0_–C_4_) and cycles (C_1_–C_4_), respectively. The realized genetic gains due to RCGS were highest in C_4_ at the two locations and combined across them.

### Changes in the mean for unselected flowering, moisture, and height traits

The effects of genomic selection on unselected flowering, moisture and height traits are shown in Table 3. On average, anthesis and silking days of the entries representing C_4_ did not increase with respect to their averages in early GS cycles (C_0,_ C_1,_ C_2_, and C_3_). They ranged from 56 d for anthesis and 57 d for silking for all genomic selection cycles, and showed good general synchrony between both flowering times. However, GS produced taller plants and ear insertions during cycles C_3_ and C_4_ than during cycles C_1_ and C_2_. Grain moisture content did not seem to have been greatly affected after the three cycles of RCGS.

**Table 3:** Means of entry and checks for traits anthesis days (AD, days), silking days (SD, days), plant height (PH, centimeter), ear height (EH, centimeter), and moisture content (MOI, %) in each cycle across the two locations

Cycle	AD		SD		PH		EH		MOI	
	Entry	Check	Entry	Check	Entry	Check	Entry	Check	Entry	Check
C_0_	56.54	56.58	57.22	57.62	250.86	249.86	130.71	136.84	17.06	17.64
C_1_	56.54	57.02	57.35	58.11	250.23	247.39	129.55	131.77	18.61	19.45
C_2_	56.60	56.82	57.05	57.71	254.22	251.15	130.86	135.54	18.51	19.03
C_3_	56.11	56.27	56.80	57.31	258.11	252.05	133.00	134.18	19.42	19.88
C_4_	56.49	56.51	57.26	57.75	260.17	255.33	136.80	137.03	17.35	17.40
LSD_0.05_ (C_0_–C_4_)	0.210	—	0.233	—	2.882	—	2.408	—	0.254	—
LSD_0.05_ (C_1_–C_4_)	0.167	—	0.173	—	2.205	—	2.306	—	0.230	—

Least significant differences at the 0.05 probability level including all cycles (LSD_0.05_ (C_0_–C_4_)) and including only the genomic selection cycle (LSD_0.05_ (C_1_–C_4_)).

The plant and ear heights for the two latest GS cycles were ∼6–10 cm higher than the plant and ear heights of the maize plants for the two earlier GS cycles (Table 3). The phenotypic variance (data not shown) of the entries for each GS cycle varied during the latest cycles (C_3_ and C_4_), and tended to decline with respect to the early cycles. Similarly, trends in broad-sense heritability are smaller in the last cycle (C_4_) than in early cycles (C_0_ and C_1_). The mean of the entries within each selection cycle, and the mean of the benchmark checks did not differ much for the different traits. For example, the check had ∼56 d to anthesis and 57 d to silking. In general, the checks had smaller plant height, taller ear height and similar grain moisture than the entries in the selection cycle.

The phenotypic correlation between the different traits and grain yield changed from different selection cycles (data not shown). Results indicated positive correlations between grain yield and days to silk ranging from 0.3 to 0.5 for the different selection cycles. Negligible and negative correlations between grain yield and plant height, anthesis days, and moisture content were found.

### Genetic diversity of the rapid cycle recombination of GS

**Figure 2:**
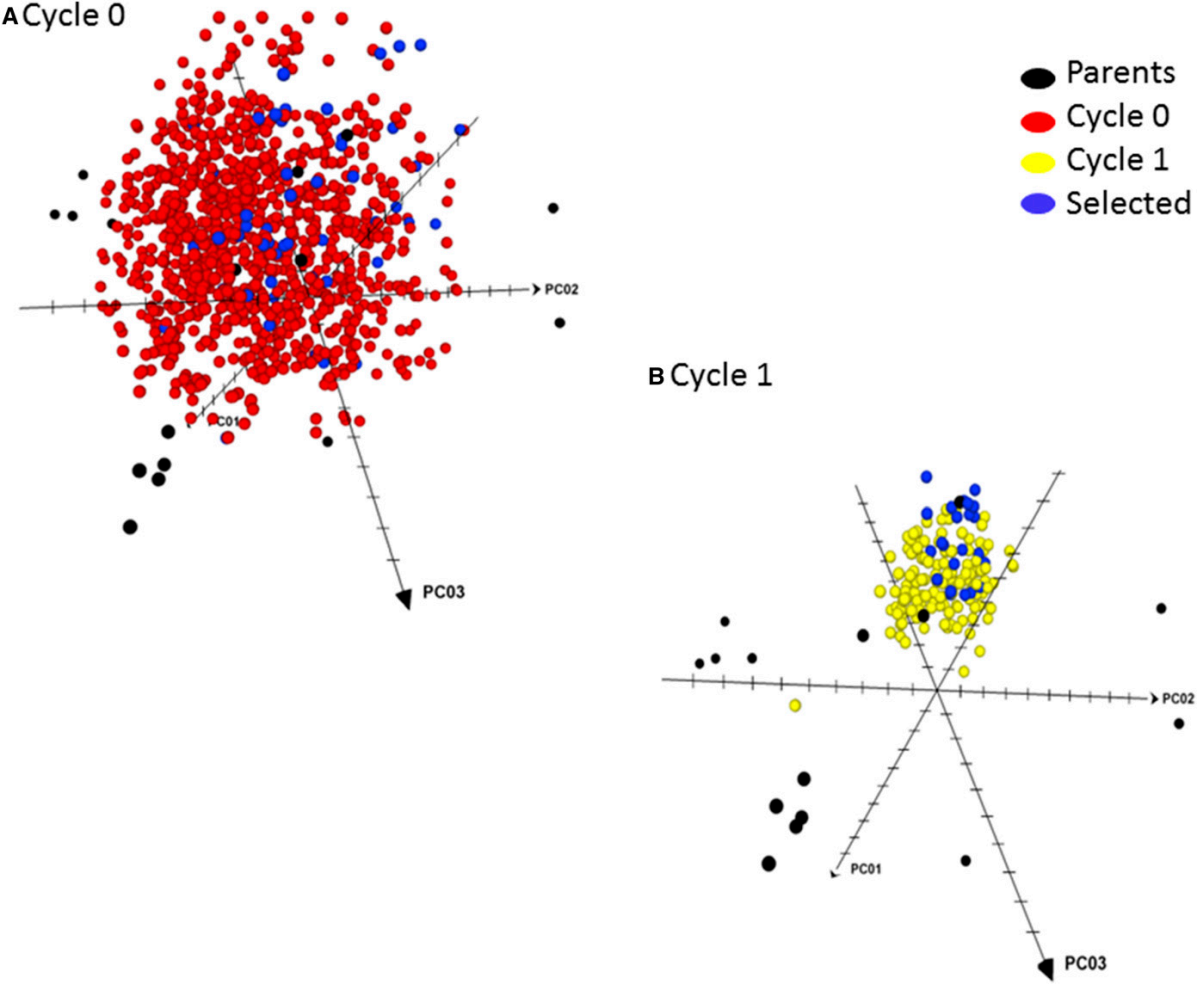
Distribution of parents, cycle C_0_ entries (A) and the selected parents, and cycle C_1_ entries (B) and the selected parents based on rapid cycling genomic selection-assisted recombination.

The diversity structure pattern including the 18 parents, the C_0_ families and the individuals selected as parents of C_1_ is displayed in Figure 2A; the selected individuals are well spread along the three dimensions, and should capture most of the diversity in the C_0_ families. The original parents, the C_1_ families and the selected individuals (that form the parents of C_2_ selection based on genomic prediction) are depicted in Figure 2B. The C_1_ families are located between dimensions 1 and 3, close to four of the original parents located in this region of the figure.

**Figure 3:**
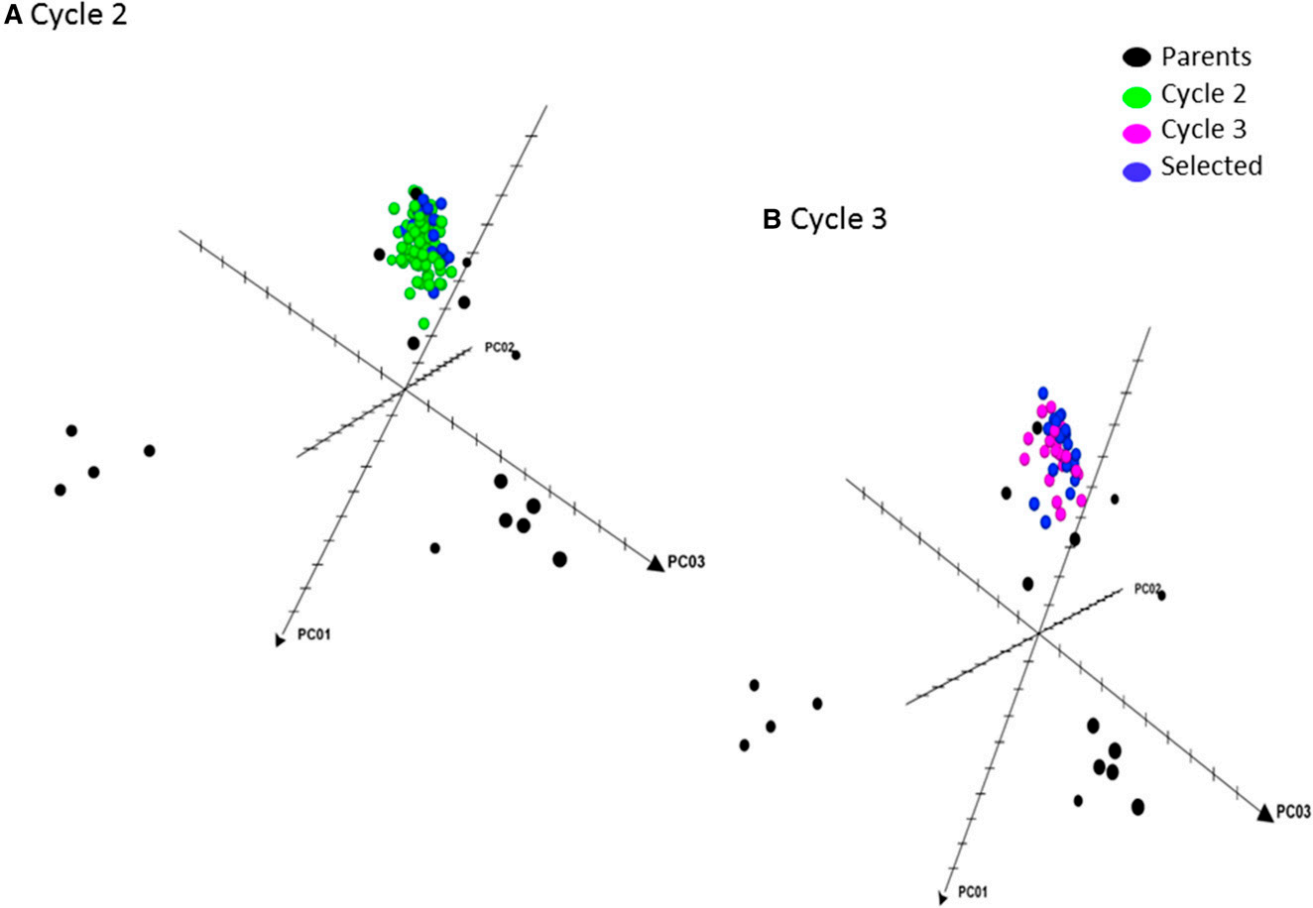
Distribution of parents, cycle C_2_ entries (A), cycle C_3_ entries (B) and the selected parents based on rapid cycling genomic selection-assisted recombination.

The original 18 parents, the C_2_ families, and the parents selected to form the next selection cycle are shown in Figure 3A. The C_2_ families and the selected parents are located between dimensions 1 and 3, clearly heading in the direction of two of the original parents located in this region of the three-dimensional figure. Finally, Figure 3B includes the original 18 parents, the C_3_ families and the selected parents that were intermated in RCGS to form C_4_. Clearly, the C_3_ families and the selected parents that form C_4_ are concentrated around the two original parents located in the upper region of the figure, between dimensions 1 and 3. However, a direct comparison between the genetic diversity of different populations (C_0_–C_4_) may be confounded by the differences in population size, and also by the different levels of inbreeding in the different selection cycles.

**Figure 4:**
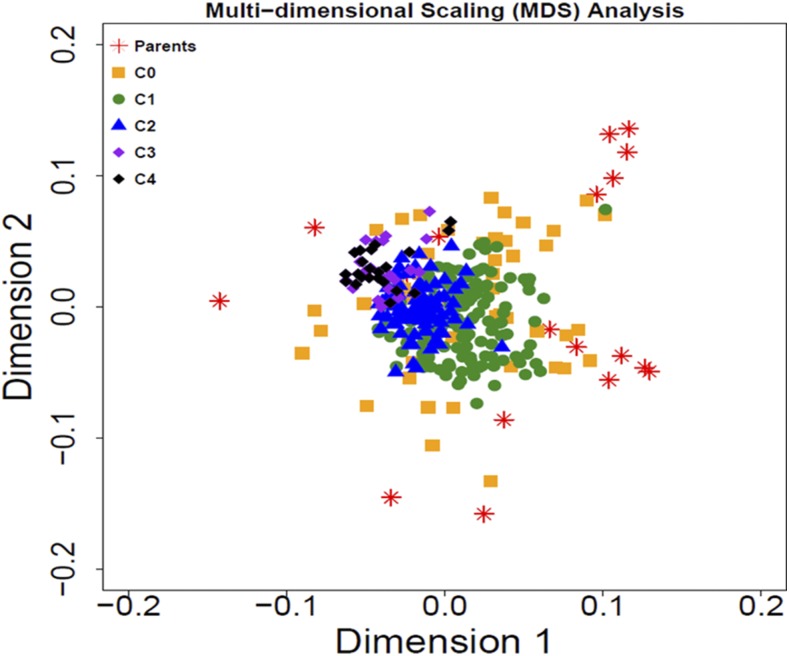
Plot of the two dimensions of the multi-dimensional scaling analysis including all samples of the original parents and families in each cycle.

Figure 4 depicts the two plot dimensions of the multidimensional scaling with the 18 parents and all families in selection cycles C_0_–C_4_. The C_3_ families and the selected parents that form C_4_ are located toward the upper left quadrant of the biplot in the same direction as one of the original parents.

**Figure 5:**
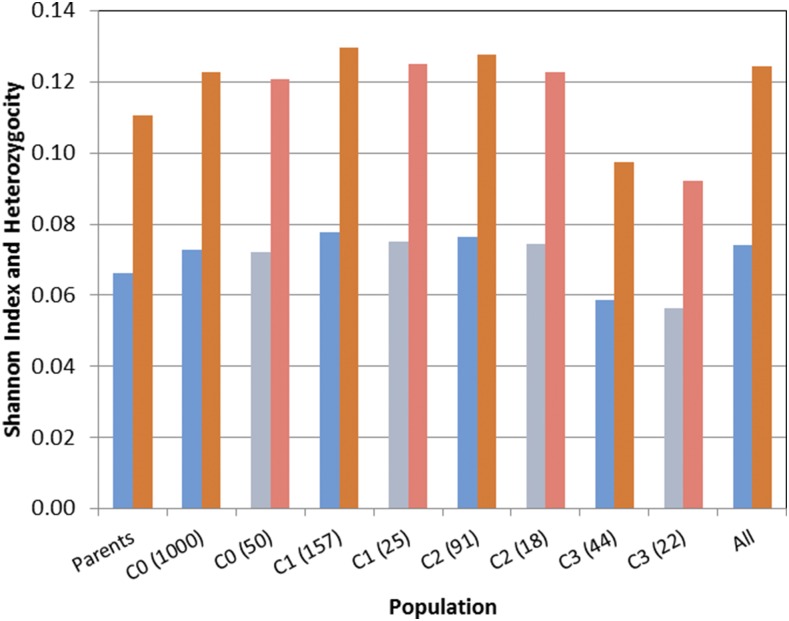
Bar-plot of the Shannon Diversity Index (blue) and heterozygosity (brown and light pink) for the original parents, individuals from cycles C_0_–C_3_ and the selected entries in light blue and light pink.

Concerning the genetic diversity of the different C_0_–C_4_ entries, Table 4 shows the values of Shannon’s Diversity Index, the heterozygosity and the number of SNPs for the 18 parents, cycles C_0_–C_4_, the selected parents from C_0_ to C_4_, along with all the entries. Results of Shannon’s Diversity Index and the heterozygosity for cycles C_0_–C_4_, along with the selected parents from C_0_ to C_4_ and all other entries, are displayed in the barplots in Figure 5. Genetic diversity did not decline in the initial cycles and the trends in Shannon’s Diversity Index and heterozygosity even showed a slight increase in cycles C1 and C2. However, there was a decrease in the three diversity measurements for the population represented by the C_3_ entries, as well as for the parents selected to form C_4_. Note that entries from C_4_ were not included because they were not genotyped.

**Table 4:** The Shannon Diversity Index, heterozygosity, and number of SNPS of the 18 original parents, the number of families in cycles C_0_–C_3_ (in parentheses), and the selected parents in C_0_–C_3_, and including all the entries

	Parents	C_0_ (1000)	C_0_ (50)	C_1_ (157)	C_1_ (25)	C_2_ (91)	C_2_ (18)	C_3_ (44)	C_3_ (22)	All Entries
Shannon’s Index	0.0661	0.0728	0.020	0.0776	0.052	0.0765	0.043	0.0588	0.063	0.0740
Heterozygosity	0.1104	0.1226	0.1208	0.1297	0.1250	0.1276	0.1228	0.0973	0.0923	0.1245
Number of SNP markers	950,248	952,825	943,344	951,390	947868	953,199	953,453	954,058	954,924	954,960

Numbers in parentheses refer to the size of the cycle population and the selected parents to form the subsequent cycle.

### Changes in the status of marker frequency

For each SNP marker, we measured the change in the allele frequency of the 18 parents’ original parents that formed C_0_, and the allele frequency of the C_3_ and C_4_ entries. Initially, allele frequency was calculated for the 18 parents, and for the entries of cycles C_3_ and C_4_; SNP markers with allele frequencies between 0.05 and 0.95 were considered polymorphic markers, whereas markers with allele frequencies <0.05 or >0.95 were considered monomorphic markers. Using these criteria, we found that from a total of 1120 SNP markers (0.117% of all markers) that changed their polymorphic/monomorphic status, 123 markers swapped the major allele frequency, 968 markers became monomorphic in C_3_–C_4_ although they were polymorphic in the 18 parents and C_0_, and only 29 markers were polymorphic in cycles C_3_ and C_4_, although they were monomorphic in the 18 parents and cycle C_0_ (Table 5). Chromosomes 1–4 had a low percent change in allele frequency (0.080–0.097%), whereas the rates of change in chromosomes 5 and 6 were 0.177, and 0.139%, respectively. Chromosomes 7–10 had a percent change in allele frequency ranging from 0.118 to 0.131%. Almost two thirds of the markers (614,663 markers, 64.37%) changed their allele frequencies without becoming monomorphic and 33.99% (324,575) of the markers changed their allele frequency by <15%.

**Table 5:** Number of SNP markers with allele swaps, number of polymorphic markers that became monomorphic and number of markers that were monomorphic and became polymorphic from [parents-C_0_] to [C_3_–C_4_]

Chromosome	Number of Allele Swaps	Number of Polymorphic to Monomorphic	Number of Monomorphic to Polymorphic	Total SNPs with Changes		Total Number of SNPs
				*N*	%	
1	12	124	7	143	0.096	148,745
2	12	105	1	118	0.102	115,152
3	7	97	1	105	0.097	108,195
4	11	64	1	76	0.080	94,716
5	20	166	9	195	0.177	110,303
6	12	91	3	106	0.139	76,450
7	7	90	2	99	0.123	80,514
8	10	86		96	0.118	81,427
9	21	71	2	94	0.130	72,355
10	11	74	3	88	0.131	67,103
Total	123	968	29	1120	0.117	954,960

Markers with changes in their frequency were clustered according to the physical distance in the map; SNPs with a physical distance <1000 bp were considered a cluster (or haplotype). A total of 88 clusters (haplotypes) of different sizes were found with three or more SNPs that changed their frequency. The distribution and size of the clusters as well the type of change are shown in Table 6. For example, chromosome 1 had one cluster with four markers that changed their frequency; this indicated that there were at most 4000 bp units where these four markers were located. Most of the changes correspond to SNP markers that became monomorphic in cycles C_3_ and C_4_ although they were polymorphic in the 18 parents and cycle C_0_. Most of the clusters were found on chromosome 5 (Table 6).

**Table 6:** Distribution and size of clusters of SNPs with changes in their polymorphic status by chromosome

Chromosome	Allele Swap				Polymorphic to Monomorphic							Monomorphic to Polymorphic			Total
	Number of SNPs				Number of SNPs							Number of SNPs			
	3	4	5	7	3	4	5	6	7	8	9	3	4	5	
1		1			3	4		2					1		11
2	1		1		4	1	1	1							9
3									1		1				2
4					2	2	1		1						6
5	2				9	3	2		1	1				1	19
6					1	3	2	1				1			8
7					2	2	1	1							6
8	2				4	2	2								10
9				1	2	1	3		1						8
10	1				4	3				1					9
Total	6	1	1	1	31	21	12	5	4	2	1	1	1	1	88

**Figure 6:**
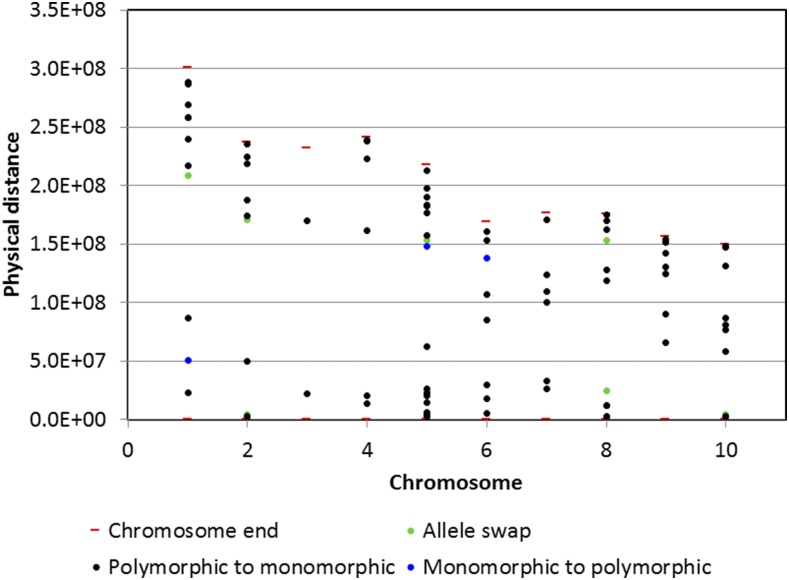
Genome location of clusters of SNPs with changes in their polymorphic status.

Figure 6 depicts the location of the clusters for each chromosome in the genome. For example, chromosome 1 has one cluster with four markers that changed their frequency (green color), one cluster of four markers that changed from monomorphic in the 18 parents and cycle C_0_ to polymorphic in cycles C_3_ and C_4_ (blue color), and three clusters with three markers, four clusters with four markers, and two clusters with six markers that changed from polymorphic in the 18 parents and cycle C_0_ to monomorphic in cycles C_3_ and C_4_ (black color). As shown in Table 6, most of the clusters with changes in their allele frequency occurred in chromosome 5.

## Discussion

Previous studies on temperate and tropical maize showed realized gains of RCGS in biparental populations (Massman *et al.* 2013; Beyene *et al.* 2015; Vivek *et al.* 2016). In this study, our results showed realized gains of RCGS in a multi-parental tropical maize population that originated from crosses of 18 CIMMYT elite tropical maize lines. From a practical breeding perspective, multi-parental populations might not be an attractive option because the mean of 18 parental lines might be lower than the mean of the best few lines that could be used in biparental crosses; however, as diversity becomes an important issue in GS, multi-parental populations offer the opportunity to maintain diversity, while still achieving rapid cycles with high realized grain yield genetic gains achieved in a shorter period of time, as found in this study. As for the decrease in genetic diversity, this is not of much concern in a short-term selection (four to five cycles), especially if the new developed lines from C_4_ are crossed with other lines for further breeding.

### Trends in the realized genetic gains of multi-parental populations for grain yield

**Figure 7:**
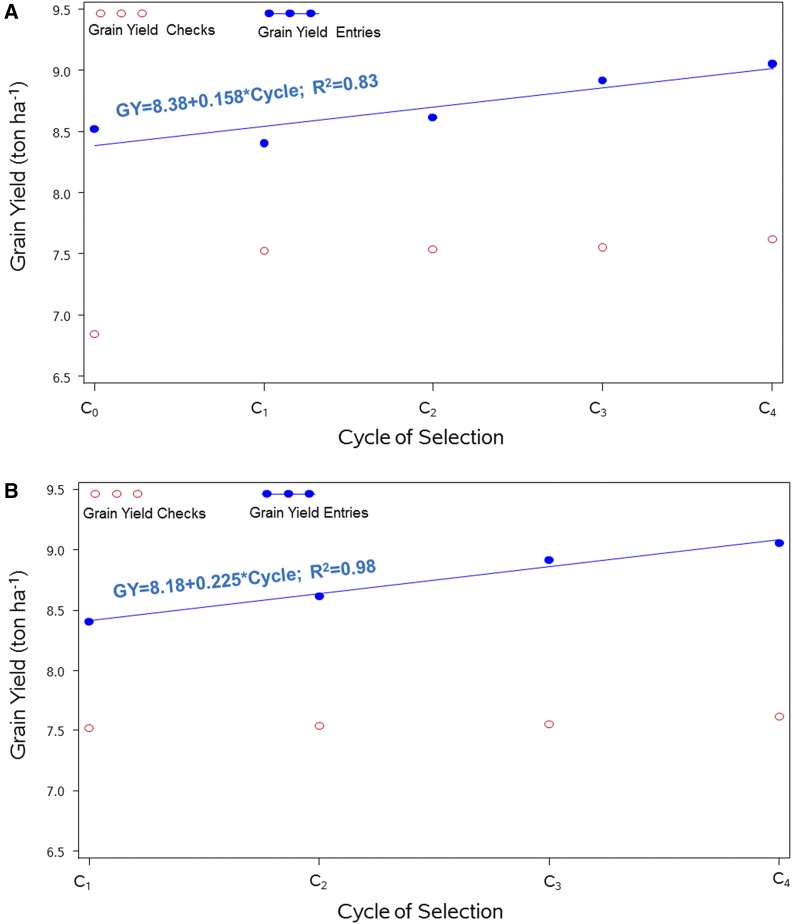
Response to selection for grain yield from the families of (A) rapid cycling recombination genomic selection for cycles C_0_, C_1_, C_2,_ C_3,_ and C_4_ and of (B) rapid cycling recombination genomic selection for cycles C_1_, C_2,_ C_3,_ and C_4_. Mean of the checks (red), and mean of the entries (blue).

The genetic gains per unit of time are given by the breeders’ equation, which is Gain = (*i×r×h*)/I, where *i* is the selection intensity, *r* is the selection accuracy, *h* is the square root of narrow-sense heritability, and *I* is the time (in years) it takes to complete a selection cycle. In this study, the gains in GY in different selection cycles were not consistent, decreasing slightly from C_0_ to C_2_, while increasing significantly from C_2_ to C_3_, and from C_3_ to C_4_. As for analyses combining the two sites, the gains in grain yield were 6.2 and 7.7% from C_0_ to C_4_ and from C_1_ to C_4_, respectively. The combined realized genetic gains reached 0.158 ton ha^−1^ per cycle for C_0_–C_4_, (Figure 7A) and 0.225 ton ha^−1^ per cycle for RCGS C_1_–C_4_ (Figure 7B).

The lower GY observed in C_1_ compared to C_0_ is explained because the best GY entries selected from C_0_ as parents of C_1_ were further intermated and selected based on flowering, plant, and ear height, etc. This also helped to broaden and maintain the genetic diversity observed in C_1_ and C_2_, which later declined in C_3_. As already mentioned, the other reason of lower GY observed in C_1_ compared to C_0_ is that the best 50 selected families (not the random selected families) were used to represent selection cycle C_0_ in the genetic gain evaluation study. The GY mean of the best 50 selected families is much higher than that value of the 50 random selected families.

In terms of the prediction models used to predict the genetic values of the entries to be selected in each genomic cycle, we used the direct genotyping-by-sequencing marker as biallelic. Since a multi-parental (not a biparental) population was used, haplotype rather than biallelic marker could have been used in order to attempt to capture the whole allelic diversity. However, the problem on how to define the length of the haplotype segment in each chromosome could impose a major drawback for using this approach; different haplotypes methods exist but none of them seems to give clear superiority in terms of genomic-enabled prediction accuracy.

### Realized genetic gains per unit of time

To compute the realized genetic gains per year (ton ha^−1^ yr^−1^), it is necessary to account for the number of cycles per year (two cycles per year in this study), and also for the time from the initial cross to the last cycle (4.5 yr from F_1_ development to harvesting the C_4_ in this study). Therefore, given that grain yield from C_1_ (8.40 ton ha^−1^) to C_4_ (9.05 ton ha^−1^) increased by 7.74%, the average genetic gain of 0.225 ton ha^−1^ per cycle (Table 2) is equivalent to 0.100 ton ha^−1^yr^−1^ [*i.e.*, (2 × 0.225)/4.5] under optimal conditions.

Masuka *et al.* (2017a) conducted a review of genetic gain studies that used conventional pedigree selection on tropical hybrid maize germplasm under optimal conditions in Sub-Saharan Africa, which gave gains of 0.109 ton ha^−1^ yr^−1^. For tropical open-pollinated maize varieties, realized genetic gains reached 0.109 ton ha^−1^ yr^−1^ in the early maturity group, and 0.079 ton ha^−1^ yr^−1^ in the intermediate-to-late group (Masuka *et al.* 2017b). Therefore, the genetic gains from the RCGS observed in the MPPs used in this study (0.100 ton ha^−1^ yr^−1^) are at the same or higher level than those observed in other studies under phenotypic selection but with a shorter breeding cycle. However, the 0.070 ton ha^−1^ yr^−1^ achieved by Beyene *et al.* (2015) in bi-parental populations is not comparable to the results of this study because the genetic gains from RCGS in biparental populations targeted managed drought environments (not optimal environments), and the RCGS in this MPP targeted optimal environments.

In this study, results obtained from MPPs in optimal environments reinforce the usefulness of GS-assisted recombination for achieving high genetic gains in GY. Although only two cycles per year were completed in this study (Beyene *et al.* 2015, completed three cycles per year in biparental populations), it is still time-efficient when compared to the 1.5 yr per selection cycle required for making testcrosses, phenotyping testcrosses, and conducting selection and recombination in conventional pedigree breeding.

### Trends in genetic diversity under rapid cycling recombination GS

There are not many reports on the influence of RCGS on genetic variance in plant breeding. In a simulation study, Jannink *et al.* (2010) were the first to caution about the possible decline in genetic variance due to RCGS. Genetic gains in GS for stem rust in wheat were reported by Rutkoski *et al.* (2015); genetic gains in GS were compared with gains in phenotypic selection and no differences were found. However, GS caused faster decline in genetic variance than phenotypic selection. Rutkoski *et al.* (2015) also found significant increases in inbreeding after one and two cycles of GS as compared with C_0_; this increase in inbreeding was significantly greater than the expected value under random genetic drift for all populations.

The above results seem to be in partial agreement with the findings of this study. The decrease in genetic diversity measured by the Shannon Diversity Index and the expected heterozygosity only occurred in RCGS in C_3_, whereas, in previous cycles, genetic diversity was very well maintained. These results may be due to the fact that C_0_ selection was initially based on GY, and then a second selection was conducted based on flowering, maturity, and other traits after intermating; this may be one of the reasons why the genetic diversity stayed at the same level as in C_0_, at least in the initial recombination cycles.

### Changes in the frequency status of markers

Results of this study show that only a few SNPs changed their polymorphic status after three cycles of GS. This result indicates that marker interaction (epistasis) may play an important role in complex traits (such as grain yield), in which phenotype is the result of the sum of the small effects of many genes. Only chromosome 5 showed a total of 19 clusters, with 16 of them having markers that became monomorphic (being polymorphic in their 18 parents and in the C_0_ training population); all the other chromosomes showed a small number of clusters with markers that became monomorphic (from their original polymorphic status) after cycles of RCGS. It is likely that some of those clusters of SNP markers with changes in their polymorphic status are related to less complex traits that were selected during C_0_ and C_1_, such as flowering time, and plant and ear height.

### Conclusions

Results described in this study are the first report of RCGS in MPPs. A realized genetic gain of ∼2% for GY with two rapid cycles per year saves time and produces efficient genetic gains overall. The decline in genetic gains from cycle C_0_ to C_1_ is because the aim of selecting parents for C_1_ was to maintain genetic diversity and the best selected families of cycle C_0_ were used as baseline for evaluating genetic gain among cycles. The realized gain achieved in this study was 0.100 ton ha^−1^ yr^−1^ when only GS cycles were considered (C_1_–C_4_). Another important finding is that genetic diversity was well controlled up to C_3_ and then declined. Although other traits were correlated with GY, they did not show any important change after three cycles of RCGS for GY. In the end, 64.3% of the markers changed their allele frequencies but never became monomorphic, and 33.99% of the markers modified their allele frequency by <15%.

The target of this study was to perform three rapid cycles per year; however, we only achieved two rapid cycles per year due to delays in the DNA preparation and genotyping turnaround time. Therefore, further studies in maize or other crops are required to confirm the promising RCGS results obtained in MPP using tropical maize lines described in this study, and examining if the length of selection cycle could be further reduced by implementing RCGS.
